# Chemical and Enantioselective Analysis of the Essential Oils from Different Morphological Structures of *Ocotea quixos* (Lam.) Kosterm

**DOI:** 10.3390/plants10102171

**Published:** 2021-10-14

**Authors:** Gianluca Gilardoni, Mayra Montalván, Marjorie Vélez, Omar Malagón

**Affiliations:** Departamento de Química, Universidad Técnica Particular de Loja (UTPL), Calle Marcelino Champagnat s/n, Loja 110107, Ecuador or gianluca.gilardoni@gmail.com (G.G.); msmontalvan@utpl.edu.ec (M.M.); mkvelez1@utpl.edu.ec (M.V.)

**Keywords:** Ishpingo, *Laurus quixos*, *Nectandra cinnamomoides*, *Mespilodaphne quixos*, American cinnamon, Ecuador

## Abstract

The traditional Ecuadorian spice Ishpingo, characterized by a strong cinnamon-like aroma, is constituted by the dry cupules of Amazonian species *Ocotea quixos*. Nevertheless, bark and leaves also present aromatic properties and are sometimes used as substitutes. In the present study, the essential oils, distilled from these morphological structures, are comparatively analyzed for their chemical and enantiomeric compositions. A total of 88 components were identified with 2 orthogonal GC columns, whereas 79, corresponding to more than 94%, were also quantified with at least 1 column. Major compounds were (*E*)-methyl cinnamate in cupules (35.9–34.2%), (*E*)-cinnamaldehyde in bark (44.7–47.0%), and (*E*)-cinnamyl acetate (46.0–50.4%) in leaves. For what concerns the enantioselective analysis, 10 chiral terpenes and terpenoids were detected, of which 6 were present as enantiomeric pairs in at least 1 essential oil, the others being enantiomerically pure. Both quantitative and enantioselective analyses were submitted to Principal Component Analysis (PCA) and Hierarchical Cluster Analysis (HCA), where their results confirmed significative difference among the three products.

## 1. Introduction

Despite that many countries are characterized by an important biodiversity all around the world, only 17 of them are known as “megadiverse”, as they account together for “two thirds of all non-fish vertebrate species and three quarters of all higher plant species” [1]. According to the UN Environment Programme, there are 17 megadiverse countries, including Ecuador. This is the reason why Ecuador is an invaluable reservoir of native and endemic botanical species, most of them unprecedented as sources of new bioactive natural products [2]. In this context, the authors have been studying for a long time the secondary metabolites of the Ecuadorian flora, as a contribution to the knowledge in its phytochemistry and phytopharmacology [3,4,5,6,7,8,9,10]. The reasons of our interest in new EOs reside in the simplicity of their obtention, the potential commercial interest, the range of biological activities (most of them unexplored), and the fact that, so far, gas chromatography-olfactometry (GC-O) and enantioselective analysis are not frequently applied to EO investigation [11,12,13,14,15,16,17,18].

However, despite new natural products and unexplored species undoubtedly arousing the highest interest from an academic point of view, many already studied plants may also need deeper investigation. That is why the present study focused on the EO of a major traditional species within the Ecuadorian flora: *Ocotea quixos*.

*Ocotea quixos* (Lam.) Kosterm. belongs to the family Lauraceae, which is also known with the accepted name *Mespilodaphne quixos* (Lam.) Rohwer and the synonyms *Laurus quixos* Lam. and *Nectandra cinnamomoides* (Kunth) Nees. *Ocotea quixos* Kosterm. ex O.C. Schmidt is a homonym [19]. Despite that some specimens have been identified also in Colombia and Peru, the very main area of dispersion corresponds to Ecuador, where it is one of the 48 species of this genus known in the country. *Ocotea quixos* is a tree, both native and cultivated, growing in the Amazonian region between 0 and 1000 m above sea level [20]. This plant is quite popular in Ecuador, where it is traditionally used as a food aroma, due to its strong similarity with cinnamon (*Cinnamomum verum* J. Presl) and to whose botanical family it belongs. The main spice, i.e., the plant derivative assuming the highest commercial importance, is constituted by the dry cupules. Other parts of the plant, such as bark and leaves, also characterized by a cinnamon-like aroma, are commonly used. Furthermore, *O. quixos* is a very slow-growing species, whose cupules can be collected only biyearly, from plants 15–20 years old. All these conditions make the production quite limited and, consequently, the spice is very expensive [21].

For what concerns the previous studies about *O. quixos* EOs, some important articles must be cited. On one hand, the chemical composition of volatile fractions, distilled from cupules, leaves and bark, were separately described [22,23,24,25]. For cupules, Bruni et al.’s study shows that the principal components detected were (*E)-*cinnamaldehyde, methylcinnamate and 1-8-cineol [22]. Sacchetti et al. found that the main components on *O. quixos* leaves were (*E*)-β-caryophyllene, (*E)*-cinnamyl acetate, sabinene, geranial and (*E)*-cinnamaldehyde [23]; whereas Valarezo et al. showed that the major components in leaves’ samples were (*E)*-cinnamyl acetate, (*E*)-methyl cinnamate and (*E*)-β-caryophyllene [24]. Finally, bark EO was studied by Noriega et al., showing that the main components were (*E)*-cinnamaldehyde, (*E*)-o-methoxy cinnamaldehyde, (*E)*-cinnamyl acetate and (*E*)-methyl cinnamate [25].

On the other hand, many biological activities, such as antimicrobial, antioxidant, antiplatelet, anti-inflammatory and larvicidal against *Aedes aegypti*, were demonstrated for some of these EOs [26,27,28,29]. More recently, we described the termiticidal and repellent activity of the leaves’ EO against *Nasutitermes corniger* [30].

The aim of the present study was to investigate the similarity among the EOs obtained from the three main morphological structures (cupules, bark and leaves). Additionally, the enantioselective analysis of the chiral components was carried out for the first time. This investigation was conducted as a component of a great governmental programme, dedicated to forest conservation and sustainable productions, through the application of non-timber forest products [31]. To the best of the authors’ knowledge, this is the first investigation of *O. quixos* EOs about these items.

## 2. Results

### 2.1. Qualitative and Quantitative Chemical Analyses

**Figure 1:**
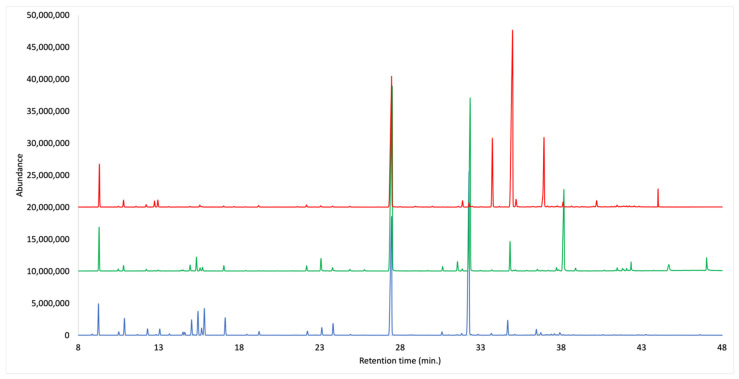
GC-MS chromatograms with DB-5ms column of the EOs from cupules, bark and leaves of *O. quixos*.

Three essential oils were obtained from dry cupules, bark and leaves of *O. quixos*, with a distillation yield by weight of 1.79%, 1.04% and 1.52%, respectively. Both qualitative and quantitative analyses were carried out by capillary gas chromatography (GC), over non-polar (polydimethylsiloxane and 5% phenyl groups) and polar (polyethylene glycol) stationary phases. In the qualitative analysis, the GC instrument was coupled with a mass spectrometer (GC-MS), whereas a flame ionization detector was applied to the quantitative one (GC-FID). A total of 88 components were identified, of which 79 were also quantified in at last one EO, with at least one column. All the quantified metabolites corresponded to more than 94% of the whole EO mass in every case. The main fraction was constituted by shikimic acid derivatives (74.5–89.1%) for all the morphological structures. They could be identified as derivatives of cinnamic acid, which is perfectly consistent with the strong cinnamon-like odor of all these EOs. In particular, (*E*)-methyl cinnamate was the major constituent of cupules’ EO (35.9–34.2%), (*E*)-cinnamaldehyde was most abundant in bark volatile fraction (44.7–47.0%), whereas leaves were dominated by (*E*)-cinnamyl acetate (46.0–50.4%). After phenylpropanoids, the second main fraction was constituted by monoterpenes. In this case, they altogether ranged from about 3% in leaves, to about 19% in cupules. The complete chemical analyses are reported in Table 1, whereas the comparative chromatograms are represented in Figure 1.

### 2.2. Enantioselective Analysis

Since the monoterpene fraction is not negligible in these EOs, an enantioselective analysis was carried out through a cyclodextrin-based capillary column. A total of 10 chiral terpenes and terpenoids were detected, of which 6 were present as enantiomeric pairs in at least one essential oil, the others being enantiomerically pure. This was the case of (1*R*,4*S*)-(-)-camphene, *(R)-(-)-*linalool and (1*R*,2*S*,6*S*,7*S*,8*S*)-(-)-α-copaene; whereas, in many other cases, the compound being enantiomerically pure is a morphological structure but presented an enantiomeric excess in the others (α-pinene, β-pinene, α-phellandrene, limonene and terpinen-4-ol). The complete enantioselective analysis is detailed in Table 2.

### 2.3. Statistical Analysis

**Figure 2:**
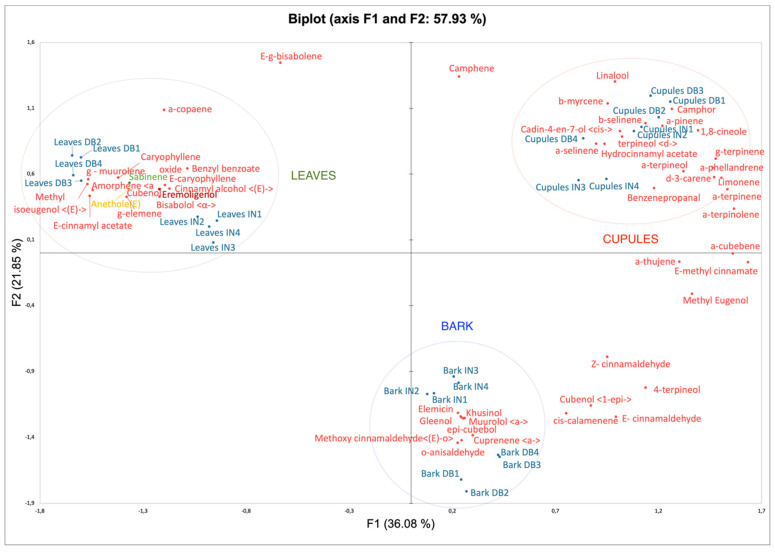
Principal Component Analysis (PCA) of leaves, bark and cupules and observations and variables Biplot Analysis.

To treat the problem more objectively, the data obtained was submitted to a PCA (Principal Component Analysis) and HCA (Hierarchical Clustering Analysis) approach. PCA based on 76 essential oil compounds resulted in two principal components: F1 (36.08%) and F2 (21.85%) with 57.9% of the total variance. The PCA showed three different groups corresponding to cupules, leaves and bark essential oils (Figure 2). These results allow to establish that, from the point of view of their EO chemical composition, the three morphological parts can be considered as different among them.

On one hand, the main contributors in leaves’ PCA were (*E*)-cinnamyl acetate, (*E*)-methyl isoeugenol, γ-muurolene, amorphene, caryophyllene oxide and cinnamyl alcohol, among others. In the case of barks, they were (*E*)-*o*-methoxy cinnamaldehyde, *o*-anisaldehyde, cuprenene, epicubebol, muurolol, elemicin, khusinol and gleenol. In cupules, they were camphor, β-selinene, α-pinene, 1,8-cineole and *cis*-cadin-4-en-7-ol. (*E*)-cinnamaldehyde and (*E*)-methyl cinnamate are principal contributors between cupules and bark (Figure 2). On the other hand, through HCA, it is possible to consider that barks and cupules’ essential oils are more similar among them than leaves’ essential oils.

## 3. Discussion

The present study demonstrated that the essential oils, distilled from the three main morphological structures of *O. quixos*, are significantly different from both the chemical and enantiomerical point of view. For this reason, instead of simply concluding that they cannot be used indifferently, it would be better to indicate the different properties and possible applications, according to what is known in literature about the respective main components. For what concerns the chemical composition, the EOs from cupules and bark are dominated by (*E*)-methyl cinnamate and (*E*)-cinnamaldehyde (although in different relative abundance), whereas the one from leaves is characterized by (*E*)-cinnamyl acetate and (*E*)-cinnamaldehyde. Since (*E*)-cinnamaldehyde is common to all these EOs, we could suppose that it exerts its properties in all cases, with the highest activity in bark and the lowest activity in leaves. According to literature, (*E*)-cinnamaldehyde presented important antifungal, antibacterial, larvicidal, repellent and anti-diabetic activities [43,44,45,46,47]. The antifungal activity was widely investigated, obtaining excellent in vitro results against *Aspergillus flavus*, *Coriolus versicolor*, *Laetiporus sulphureus*, *Saccharomyces cerevisiae*, *Aspergillus parasiticus*, *Aspergillus niger*, *Candida albicans*, *Collectotrichum gloeosporioides*, *Rhizoctonia solani*, *Fusarium solani* and *Ganoderma austral*, most of which are pathogenic. Furthermore, this molecule resulted remarkably active against fluconazole-resistant strains of *C. albicans*. The (*E*)-cinnamaldehyde antifungal mechanism is supposed to be varied, probably interfering with the cell wall biosynthesis, the membrane functions and inhibiting important enzymes [43]. On the other hand, the antibacterial activity has been mainly described for (*E*)-cinnamaldehyde-dominated EOs, where our molecule was confirmed to be the active component. Both Gram-positive and Gram-negative bacteria resulted to be sensitive to (*E*)-cinnamaldehyde, which is quite interesting since usually Gram-negative bacteria are more resistant to antibiotics. The main reason for this property is the ability of (*E*)-cinnamaldehyde to inhibit porins (membrane proteins), affecting the osmotic equilibrium of the cell and increasing its vulnerability. However, many mechanisms other than osmotic interference have been confirmed, such as ATPase inhibition, cell division inhibition, mobility inhibition, biofilm formation and anti-quorum sensing effect. However, the most important property is probably the synergic antibacterial effect, resulting in the increased efficacy of classical antibiotics when administrated in addition to (*E*)-cinnamaldehyde [44]. Another important biological activity of this metabolite is its toxicity against *Culex pipiens pallens*, a subspecies of common mosquito, and *Aedes aegypti* larvae. These activities, that can be exerted respectively by fumigation and irroration, open the way to possible applications in the Amazonian Forest, where *O. quixos* is cultivated and biting pests are responsible for spreading serious diseases [45,46]. Furthermore, we must consider the anti-diabetic properties of (*E*)-cinnamaldehyde that was widely assayed in animal models, showing a variety of physiological effects on different tissues and organs, all converging toward the control of glycemia [47]. About (*E*)-methyl cinnamate, many biological activities such as antibacterial, antifungal, antispasmodic, myorelaxant and anti-inflammatory have been described. However, the most recent and interesting property is probably its activity against pre-osteoblast survival, migration and differentiation, which suggests possible applications to the treatment of many bone diseases [48]. Finally, to speculate over possible activities for our leaves’ EO, the properties of (*E*)-cinnamyl acetate must be considered. Despite that this compound is quite less investigated in respect to the other cinnamic derivatives, the main biological activity is an excellent anti-inflammatory capacity. The pharmacological mechanism is the suppression of nitric oxide production [49]. All these observations, together with the statistical results previously described, can be considered quite representative of the different EOs obtained from *O. quixos*. In fact, their quali-quantitative chemical compositions are very consistent with those presented in literature by other authors.

**Figure 3:**
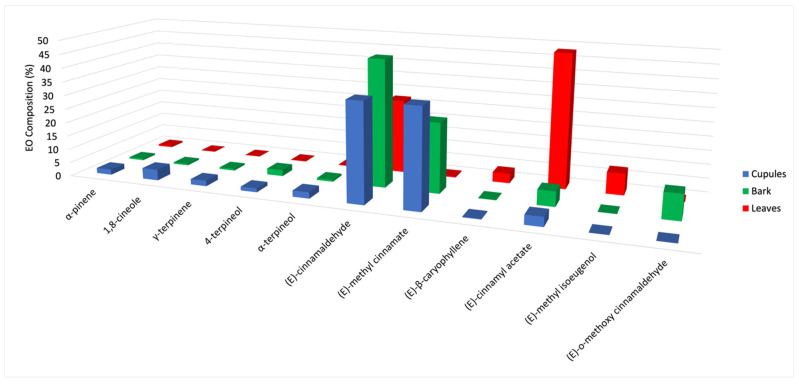
Simplified comparative histogram of the mean quantitative analyses for major components (>2% for at least one EO).

The chemical analysis of the three essential oils substantially confirms the analytical results present in literature for the volatile fractions of *O. quixos* [22,23,24,25]. Furthermore, an enantioselective evaluation of the terpene components was carried out here for the first time. For what concerns the chemical composition, a simplified representation is shown in Figure 3.

In this graph, only those constituents, whose abundance is >2% by at least one column, are represented. From a qualitative point of view, a fact immediately stood up: the relative abundance of the three main components appeared dramatically different within the samples. On one hand, the main component changed in each EO, being (*E*)-methyl cinnamate dominant in cupules, (*E*)-cinnamaldehyde in bark and (*E*)-cinnamyl acetate in leaves. On the other hand, (*E*)-methyl cinnamate is quite absent where (*E*)-cinnamyl acetate is overwhelming (leaves), whereas the opposite is observed in cupules and bark. These observations could induce to think that the three EOs are basically different and, subsequently, the three morphological structures are not so similar in their EO composition to be used as spices or equivalent sources of a cinnamon-like aroma.

**Figure 4:**
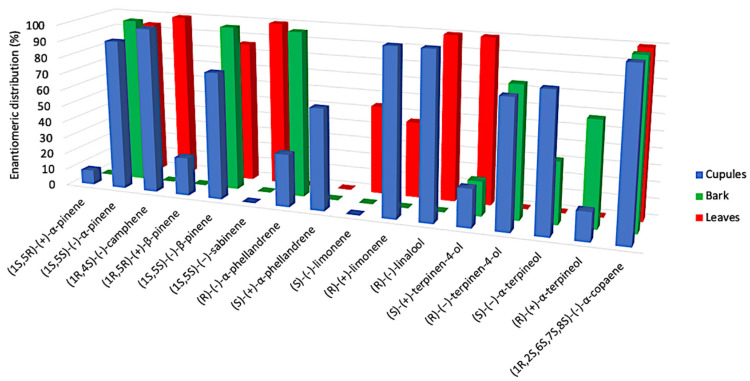
Comparative histogram of the enantioselective analysis for the three EOs.

For what concerns the enantioselective analysis, a quite interesting phenomenon aroused. In fact, where a chiral compound was detected in more than one EO, the enantiomeric distribution was not respected everywhere. This is the case of α-phellandrene, limonene, terpinene-4-ol and α-terpineol, whose major enantiomer is levorotatory in a certain morphological structure and dextrorotatory in another. In this respect, the enantiomer composition of limonene is the most peculiar, since it appears to be enantiomerically pure in cupules (dextrorotatory), undetected in barks and almost racemic in leaves (with the levorotatory isomer as the major one). Anyway, the sum of analyzed chiral components corresponded to 8.3%, 4.3% and 3.2% of the total EO for cupules, bark and leaves, respectively. These amounts are quite small to imagine a great influence of the enantiomeric distribution on the total aroma profile. Actually, none of these chiral terpenes is a major constituent of any EO. These results are represented in Figure 4.

## 4. Materials and Methods

### 4.1. Instruments and Chemicals

Essential oil qualitative and enantioselective analyses were performed using an Agilent Technologies GC-MS system (GC 6890N, Autoinjector model 7683). The device was coupled to a mass spectrometry detector (MSD) model 5973 INERT from Agilent Technologies (Santa Clara, CA, USA). The MSD was programmed in SCAN mode (range 40–350 *m*/*z*) through an electron ionization (EI) source at 70 eV. The transfer line temperature was set at 280 °C, whereas the MS ion source was kept at 200 °C. For quantitative analyses, a common flame ionization detector (FID) was used instead of MSD. The FID worked with a mixture of hydrogen and air at the flow of 30 mL/min and 300 mL/min, respectively. The detector was operated at 250 °C. The non-polar column was composed by a 5% phenyl methylpolysiloxane stationary phase (DB-5ms from Agilent Technologies, 30 m long, 0.25 mm internal diameter and 0.25 μm film thickness), while the polar column was fit with a polyethylene glycol phase (HP-INNOWax from Agilent Technologies, 30 m × 0.25 mm × 0.25 μm). The enantioselective analysis was achieved into 30% diethyl-*tert*-butyldimethylsilyl-β-cyclodextrin capillary column on PS-086 as the chiral selector. The column was 25 m × 250 μm internal diameter × 0.25 μm phase thickness and was purchased from Mega, MI, Italy. Helium purity grade (Indura, Guayaquil, Ecuador) was used as gas for GC analyses at a 1 mL/min flow rate. The C_10_–C_25_ *n*-alkanes mixture, the internal standard for GC analysis (*n*-nonane) and all analytical grade solvents (purity >99%) were acquired from Sigma-Aldrich. Isopropyl caproate, as calibration standard for GC-FID, was produced and purified in the authors’ laboratory at 98.8% purity.

Statistical analyses were carried out with XLSTAT (Version 2021.3.1.1155, Addinsoft, Paris, France) and MS Excel (Version 16.51, Redmond, WA, USA).

### 4.2. Plant Material

Dry cupules, bark and leaves of *O. quixos* were purchased in 2019 from the Chankuap Foundation (Soasti, 10 de Agosto y Tarqui, Morona Santiago, Macas, Ecuador). All vegetal materials were obtained from cultivated plants, grown by the foundation’s rural providers in Morona-Santiago province (Amazonia) of Ecuador.

### 4.3. EO Distillation and Sample Preparation

An amount of 100 g of plant material from each morphological structure was distilled in duplicate, through a laboratory-scale glassy Dean-Stark apparatus, obtaining six EO samples. Each distillation was carried out for 2 h, affording an organic phase that spontaneously separated from water. After separation, the EOs were dried over anhydrous sodium sulphate and kept in darkness, at −15 °C, until use.

For all chemical and enantioselective analyses, the samples were prepared as previously described in literature [12]. In this process, two samples were prepared from each EO, finally counting on a total of 12 analytical samples, that were directly injectable in GC.

### 4.4. Chemical Qualitative Analysis

In qualitative analyses, a representative analytical sample of each EO was submitted to GC-MS over both polar and non-polar stationary phases. With each column, the *n*-alkanes homologous series was also injected. The GC conditions with DB-5ms were as follows: 50 °C as initial constant temperature for 5 min, subsequently, a gradient of 3 °C/min until 155 °C, followed by an additional gradient of 15 °C/min until 250 °C. The last temperature was maintained constant for 2 min. With the INNOWax column, the thermal program was identical, except for the last temperature that was fixed at 230 °C. The injector was set at 250 °C and operated in split mode (40:1) with both columns. As usual, all the analytes were identified, with both columns, by comparing each EI-MS spectra and the respective linear retention index (LRI) with data from literature (see Table 1). The LRIs were calculated according to Van Den Dool and Kratz [50].

### 4.5. Chemical Quantitative Analysis

The quantitative analyses were carried out injecting the 12 analytical samples in both columns and expressing the percent results as mean values and standard deviation. The GC configuration and analytical method were the same as the qualitative analysis. The quantification was conducted by means of a relative response factor (RRF) that was calculated based on each analyte’s combustion enthalpy [51,52]. A calibration curve was built for each column as previously described in literature [12], using isopropyl caproate as calibration standard and *n*-nonane as internal standard.

### 4.6. Enantioselective Analysis

The enantiomeric composition was evaluated by GC-MS, injecting a representative sample for each EO. The MSD configuration and injection condition were the same as the qualitative analysis, whereas the thermal program was as follows: 50 °C for 5 min, then a gradient of 2 °C/min until 220 °C, finally, 220 °C for 5 min. The enantiomers were determined by injecting enantiomerically pure standards in the same column and applying identical instrumental conditions.

### 4.7. Statistical Analysis

The different observations (chemical quantitative and enantioselective) were analyzed through Principal Component Analysis (PCA) and Hierarchical Cluster Analysis (HCA) to assess the differences in chemical composition of essential oil among different parts of the plant and the different GC-FID analysis performed (DB-5ms and HP-INNOWax). PCA used correlation matrix (Pearson matrix). HCA was performed with Euclidean distance and Ward’s minimum variance method. Standard deviation (σ) was calculated using MS Excel.

## 5. Conclusions

In conclusion, each one of the three main dry morphological structures of *O. quixos* produced, by hydrodistillation, an EO in similar and very good yields. On one hand, the statistical analyses demonstrated that the three EOs must be considered different from both the chemical and enantiomeric points of view. On the other hand, despite the analyzed fraction of chiral constituents which did not exceed 8% of the whole EO, some monoterpenes (α-pinene, β-pinene and limonene) are known to be characterized by a quite low threshold odor concentration and a different aroma for the two enantiomers. Consequently, the three morphological structures can certainly be used as spices or sources of EOs, however, these three products cannot be commercially considered as equivalent. Since, currently, the three morphological structures are almost indifferently used as cinnamon-like aromas, these results could open the way to a more rational and economically sustainable production of cinnamon-like aromas in the Amazonian region of Ecuador.

## Figures and Tables

**Table 1:** Chemical analysis of the EOs from the three morphological structures, with both DB-5ms and HP-INNOWax columns.

No.	Compounds	DB–5ms									HP–INNOWax								
		Retention Indices			Cupules		Bark		Leaves		Retention Indices			Cupules		Bark		Leaves	
		LRI ^1^	LRI ^2^	Ref.	%	σ	%	σ	%	σ	LRI ^1^	LRI ^2^	Ref.	%	σ	%	σ	%	σ
1	α–thujene	924	924	[32]	0.4	0.12	0.3	0.05	trace	–	1024	1027	[33]	0.2	0.19	0.2	0.05	trace	–
2	α–pinene	930	932	[32]	2.0	0.57	0.7	0.10	0.5	0.07	1019	1028	[34]	1.9	0.54	0.6	0.09	0.7	0.1
3	camphene	947	946	[32]	0.1	0.03	trace	–	0.1	0.01	1060	1069	[33]	0.1	0.04	trace	–	trace	–
4	benzaldehyde	960	952	[32]	0.3	0.5	0.3	0.20	0.1	0.05	1535	1534	[35]	1.3	0.09	0.3	0.05	0.4	0.07
5	sabinene	971	969	[32]	trace	–	trace	–	0.4	0.1	1121	1122	[33]	0.1	0.04	0.1	0.03	0.6	0.11
6	β–pinene	975	974	[32]	0.2	0.28	trace	–	0.6	0.11	1107	1110	[33]	0.8	0.23	0.1	0.03	0.7	0.11
7	β–myrcene	989	988	[32]	0.3	0.07	trace	–	trace	–	1167	1167	[36]	0.3	0.17	trace	–	0.1	0.01
8	α–phellandrene	1009	1002	[32]	0.3	0.09	0.1	0.02	trace	–	1164	1168	[33]	0.3	0.07	0.1	0.02	trace	–
9	δ–3–carene	1011	1011	[32]	0.4	0.11	0.1	0.02	trace	–	1146	1147	[33]	0.3	0.08	0.1	0.02	trace	–
10	α–terpinene	1016	1014	[32]	1.5	0.41	0.7	0.13	trace	–	1178	1179	[36]	1.4	0.35	0.6	0.12	0.1	0.02
11	*p*–cymene	1022	1020	[32]	0.1	0.01	trace	–	trace	–	1272	1270	[33]	2.3	0.37	1.2	0.27	trace	–
12	*o*–cymene	1027	1022	[32]	2.2	0.4	1.3	0.29	trace	–	1275	1281	[37]	trace	–	trace	–	trace	–
13	limonene	1028	1024	[32]	1.0	0.26	0.5	0.10	0.2	0.03	1198	1198	[33]	0.9	0.21	0.4	0.08	0.2	0.04
14	1,8–cineole	1031	1026	[32]	4.0	0.56	0.6	0.17	trace	–	1209	1211	[33]	3.9	0.46	0.6	0.16	0.1	0.02
15	γ–terpinene	1057	1054	[32]	2.0	0.44	0.6	0.12	0.1	0.01	1245	1254	[34]	1.9	0.4	0.6	0.11	0.1	0.02
16	α–terpinolene	1087	1086	[32]	0.1	0.02	0.1	0.01	trace	–	1282	1282	[33]	0.1	0.04	0.1	0.01	trace	–
17	α–thujone	1099	1101	[32]	trace	–	trace	–	trace	–	1424	1423	[33]	trace	–	trace	–	trace	–
18	linalool	1100	1095	[32]	0.9	0.01	0.1	0.04	0.3	0.06	1569	1556	[34]	1.1	0.07	0.1	0.03	0.4	0.07
19	camphor	1154	1141	[32]	0.1	0.01	trace	–	trace	–	1516	1515	[33]	0.1	0.01	trace	–	trace	–
20	benzenepropanal	1161	1162	[38]	0.7	0.12	0.6	0.09	0.1	0.03	1797	1793	[39]	0.8	0.09	trace	–	0.3	0.01
21	δ–terpineol	1177	1162	[32]	0.1	0.07	trace	–	trace	–	1689	1679	[33]	0.1	0.04	trace	–	trace	–
22	4–terpineol	1180	1174	[32]	1.4	0.03	2.4	0.63	0.1	0.04	1615	1612	[34]	1.4	0.07	2.2	0.56	0.2	0.03
23	α–terpineol	1195	1186	[32]	2.2	0.07	0.7	0.17	0.1	0.04	1714	1718	[36]	2.2	1.07	0.7	0.11	0.2	0.03
24	methyl chavicol	1197	1195	[32]	trace	–	trace	–	trace	–	1679	1671	[33]	trace	–	trace	–	trace	–
25	(*Z*)–cinnamaldehyde	1218	1217	[32]	0.2	0.07	0.2	0.04	0.1	0.01	1910	1879	[33]	0.3	0.02	0.4	0.04	0.1	0.09
26	o–anisaldehyde	1237	1239	[32]	trace	–	0.1	0.02	trace	–	1979	–	–	trace	–	0.3	0.02	trace	–
27	(*E*)–cinnamaldehyde	1274	1267	[32]	33.8	1.63	44.7	1.63	25.1	0.91	2069	2033	[33]	35.4	2.9	47.0	1.41	28.9	0.99
28	(*E*)–anethole	1279	1283	[32]	trace	–	trace	–	0.2	0.01	1837	1826	[33]	trace	–	trace	–	0.1	0.01
29	(*E*)–cinnamyl alcohol	1299	1303	[32]	trace	–	trace	–	0.2	0.05	–	–	–	–	–	–	–	–	–
30	carvacrol	1306	1298	[32]	trace	–	trace	–	trace	–	1875	2211	[33]	trace	–	trace	–	trace	–
31	(*Z*)–methyl cinnamate	1310	1299	[32]	trace	–	trace	–	trace	–	1974	2075	[33]	0.1	0.04	trace	–	trace	–
32	bicycloeleme	1324	1336	[32]	trace	–	trace	–	0.1	0.07	1475	1488	[33]	trace	–	trace	–	trace	–
33	α–cubebene	1346	1348	[32]	0.5	0.01	0.4	0.06	trace	–	1459	1460	[33]	0.5	0.04	0.3	0.05	0.1	0.19
34	eugenol	1359	1356	[32]	0.1	0.09	trace	–	trace	–	–	–	–	–	–	–	–	–	–
35	isoledene	1362	1374	[32]	0.1	0.02	trace	–	trace	–	–	–	–	–	–	–	–	–	–
37	hydrocinnamyl acetate	1375	1366	[32]	trace	–	trace	–	trace	–	1959	1944	[40]	trace	–	trace	–	trace	–
38	α–copaene	1374	1374	[32]	0.3	0.05	0.1	0.01	0.6	0.06	1479	1475	[41]	0.3	0.03	0.1	0.03	0.6	0.06
39	(*E*)–methyl cinnamate	1381	1376	[32]	35.9	3.59	26.2	1.90	trace	–	2097	2075	[33]	34.2	3.69	24.4	2.01	0.6	0.16
40	β–elemene	1389	1389	[32]	0.2	0.35	trace	–	0.1	0.14	1578	1591	[33]	trace	–	trace	–	trace	–
41	methyl eugenol	1400	1403	[32]	0.1	0.05	0.1	0.01	trace	–	2047	2023	[34]	0.1	0.01	0.1	0.01	0.1	0.02
42	(*E*)–β–caryophyllene	1418	1417	[32]	0.4	0.05	0.2	0.01	7.0	0.32	1585	1599	[33]	trace	–	0.1	0.03	trace	–
43	γ–elemene	1421	1434	[32]	trace	–	trace	–	0.1	0.01	1631	1639	[33]	trace	–	trace	–	0.1	0.06
44	β–duprezianene	1425	1421	[32]	trace	–	trace	–	trace	–	1771	–	–	trace	–	trace	–	trace	–
45	*trans*–α–bergamotene	1439	1432	[32]	trace	–	trace	–	trace	–	1578	1576	[33]	0.4	0.22	trace	–	trace	–
46	(*Z*)–β–farnesene	1447	1440	[32]	0.1	0.01	trace	–	trace	–	–	–	–	–	–	–	–	–	–
47	(*E*)–cinnamyl acetate	1446	1443	[32]	3.2	0.33	5.2	0.42	46.0	1.88	2169	2153	[40]	3.6	0.26	5.6	0.52	50.4	1.32
48	α–humulene	1454	1452	[32]	0.2	0.02	trace	–	0.4	0.31	1658	1667	[33]	0.1	0.09	trace	–	0.7	0.07
49	(*E*)–β–farnesene	1459	1454	[32]	trace	–	trace	–	trace	–	1640	1664	[33]	0.1	0.03	trace	–	trace	–
50	*trans*–cadina–1(6),4–diene	1470	1475	[32]	trace	–	0.1	0.01	0.1	0.03	–	–	–	trace	–	trace	–	trace	–
51	γ–muurolene	1470	1478	[32]	trace	–	0.2	0.03	0.1	0.01	1683	1690	[33]	trace	–	trace	–	0.1	0.01
52	γ–himachalene	1479	1481	[32]	trace	–	trace	–	0.4	0.35	1705	1709	[33]	trace	–	trace	–	0.1	0.01
53	germacrene D	1480	1480	[32]	0.1	0.01	0.1	0.03	trace	–	1698	1708	[33]	0.1	0.05	trace	–	0.1	0.01
54	α–amorphene	1482	1483	[32]	trace	–	trace	–	0.1	0.01	1678	1693	[33]	trace	–	trace	–	0.1	0.01
55	(*E*)–methyl isoeugenol	1488	1491	[32]	trace	–	trace	–	7.5	0.4	2211	–	–	trace	–	trace	–	8.2	0.27
56	trans–muurola–4(14),5–diene	1489	1493	[32]	trace	–	trace	–	trace	–	1700	–	–	trace	–	trace	–	trace	–
57	β–selinene	1491	1489	[32]	1.0	0.1	trace	–	trace	–	1705	1717	[33]	1.2	0.62	trace	–	trace	–
58	*epi*–cubebol	1494	1493	[32]	trace	–	0.1	0.01	trace	–	–	–	–	–	–	–	–	–	–
59	α–cuprenene	1499	1505	[32]	trace	–	0.2	0.04	trace	–	1760	–	–	trace	–	trace	–	trace	–
60	α–selinene	1503	1498	[32]	0.4	0.05	trace	–	trace	–	1711	1725	[33]	0.2	0.32	trace	–	trace	–
61	β–bisabolene	1508	1505	[32]	trace	–	trace	–	0.1	0.02	1723	1728	[33]	0.2	0.03	trace	–	trace	–
62	*trans*–β–guaiene	1513	1502	[32]	trace	–	trace	–	trace	–	–	–	–	0.1	0.01	trace	–	trace	–
63	δ–cadinene	1519	1522	[32]	0.1	0.15	0.3	0.02	trace	–	1750	1758	[33]	0.4	0.08	0.3	0.02	0.3	0.34
64	γ–cadinene	1524	1513	[32]	0.1	0.07	trace	–	trace	–	1747	1763	[33]	trace	–	trace	–	trace	–
65	*cis*–calamenene	1527	1528	[32]	trace	–	0.1	0.01	trace	–	1827	1835	[33]	0.1	0.01	0.1	0.01	trace	–
66	(*E*)–*o*–methoxy cinnamaldehyde	1529	1527	[32]	trace	–	8.8	4.25	trace	–	2499	–	–	trace	–	9.5	3.62	trace	–
67	α–calacorene	1533	1544	[32]	trace	–	trace	–	trace	–	–	–	–	–	–	–	–	–	–
68	(*E*)–γ–bisabolene	1540	1529	[32]	0.5	0.08	trace	–	0.6	0.02	1753	1745	[33]	0.5	0.09	trace	–	0.6	0.1
69	γ–dehydro–*ar*–himachalene	1541	1530	[32]	trace	–	trace	–	trace	–	–	–	–	–	–	–	–	–	–
70	trans–cadina–1,4–diene	1547	1533	[32]	0.3	0.06	trace	–	trace	–	–	–	–	–	–	–	–	–	–
71	elemicin	1548	1555	[32]	trace	–	0.2	0.06	trace	–	–	–	–	–	–	–	–	–	–
72	germacrene B	1551	1559	[32]	trace	–	trace	–	trace	–	1816	1824	[33]	trace	–	trace	–	0.1	0.01
73	β–calacorene	1556	1564	[32]	trace	–	trace	–	trace	–	–	–	–	–	–	–	–	–	–
74	spathulenol	1576	1577	[32]	trace	–	trace	–	0.2	0.02	–	–		–	–	–	–	–	–
75	caryophyllene oxide	1583	1582	[32]	0.1	0.04	trace	–	1.2	0.19	1980	1986	[33]	0.1	0.04	trace	–	0.7	0.53
76	gleenol	1596	1586	[32]	trace	–	0.1	0.01	trace	–	–	–	–	–	–	–	–	–	–
77	1–*epi*–cubenol	1598	1627	[32]	0.1	0.05	0.3	0.02	trace	–	2078	2088	[33]	0.2	0.05	0.4	0.03	trace	–
78	guaiol	1606	1600	[32]	0.1	0.02	trace	–	trace	–	–	–	–	–	–	–	–	–	–
79	1,10–di–*epi*–cubenol	1607	1619	[32]	trace	–	trace	–	0.1	0.01	–	–	–	–	–	–	–	–	–
80	eremoligenol	1623	1629	[32]	trace	–	trace	–	0.1	0.01	–	–	–	–	–	–	–	–	–
81	10–*epi*–γ–eudesmol	1626	1622	[32]	trace	–	0.1	0.05	trace	–	–	–	–	–	–	–	–	–	–
82	*cis*–cadin–4–en–7–ol	1637	1635	[32]	0.1	0.04	trace	–	trace	–	1628	–	–	trace	–	trace	–	trace	–
83	α–muurolol	1643	1644	[32]	0.1	0.02	0.2	0.01	trace	–	–	–	–	–	–	–	–	–	–
84	α–cadinol	1657	1652	[32]	trace	–	trace	–	trace	–	–	–	–	–	–	–	–	–	–
85	7–*epi*–α–eudesmol	1667	1662	[32]	0.2	0.03	0.2	0.04	trace	–	–	–	–	–	–	–	–	–	–
86	khusinol	1675	1679	[32]	trace	–	0.5	0.11	trace	–	–	–	–	–	–	–	–	–	–
87	α–bisabolol	1691	1685	[32]	trace	–	trace	–	0.1	0.01	–	–	–	–	–	–	–	–	–
88	benzyl benzoate	1774	1769	[42]	0.2	0.04	trace	–	1.0	0.14	–	–	–	–	–	–	–	–	–
**Hydrocarbon monoterpenes**					**10.6**		**4.4**		**1.9**					**10.6**		**4.1**		**2.5**	
**Oxygenated monoterpenes**					**8.7**		**3.8**		**0.5**					**8.8**		**3.6**		**0.9**	
**Hydrocarbon sesquiterpenes**					**4.3**		**1.8**		**9.7**					**4.2**		**0.9**		**2.9**	
**Oxygenated sesquiterpenes**					**0.7**		**1.4**		**1.7**					**0.3**		**0.4**		**0.7**	
**Shikimic acid derivatives**					**74.5**		**86.4**		**80.3**					**75.8**		**87.6**		**89.1**	
**Total**					**98.8**		**97.8**		**94.1**					**99.7**		**96.6**		**96.1**	

^1^ Calculated LRI; ^2^ Reference LRI according to Ref.; Trace is <0.1%. σ: Standard deviation.

**Table 2:** Enantioselective analysis of the EOs with a 2,3-diethyl-6-*tert*-butyldimethylsilyl-β-cyclodextrin-based column.

Enantiomers	LRI ^1^	Cupules		Bark		Leaves	
		Enantiomer Ratio (%)	*e.e.* ^2^ (%)	Enantiomer Ratio (%)	*e.e.* ^2^ (%)	Enantiomer Ratio (%)	*e.e.* ^2^ (%)
*(1S,5R)-(+)-α*-pinene	935	8.8	82.4	u/t	100.0	5.8	88.3
*(1S,5S)-(-)-α-*pinene	943	91.2		100.0		94.2	
(1*R*,4*S*)-(-)-camphene	960	100.0	100.0	u/t	–	100.0	100.0
*(1R,5R)-(+)-β*-pinene	996	23.1	53.8	u/t	100.0	14.1	71.8
*(1S,5S)-(-)-β*-pinene	999	76.9		100.0		85.9	
*(1S,5S)-(−)-*sabinene	1000	u/t	–	u/t	–	100.0	100.0
*(R)-(-)-α*-phellandrene	1032	31.8	29.4	100.0	100.0	u/t	–
*(S)-(+)-α*-phellandrene	1034	61.2		u/t		u/t	
*(S)-(-)-*limonene	1055	u/t	100.0	u/t	–	53.9	7.8
*(R)-(+)-*limonene	1061	100.0		u/t		46.1	
*(R)-(-)-*linalool	1202	100.0	100.0	u/t	–	100.0	100.0
*(S)-(+)-*terpinen-4-ol	1273	23.1	53.8	20.9	58.2	100.0	100.0
*(R)-(−)*-terpinen-4-ol	1275	76.9		79.1		u/t	
*(S)-(−)-α*-terpineol	1309	82.7	65.4	37.1	25.8	u/t	–
*(R)-(+)-α-*terpineol	1315	17.3		62.9		u/t	
(1*R*,2*S*,6*S*,7*S*,8*S*)-(-)-α-copaene	1379	100.0	100.0	100.0	100.0	100.0	100.0

^1^ LRI = Calculated linear retention indices; ^2^ *e.e* = enantiomeric excess; u/t = undetected or detected as a trace.

## Data Availability

Raw data are available from the authors.
